# KOMBREAD, Kombucha as a Functional Starter for Sourdough Bread: Physicochemical Properties, Microbiological Characterization, and Quality Evaluation

**DOI:** 10.1002/fsn3.72433

**Published:** 2026-09-30

**Authors:** Manuel Chinello, Ragy Nader Ragheb Tawfik, Elisa Canazza, Chiara Nadai, Frederico Augusto Ribeiro de Barros, Giovanna Lomolino, Anna Lante, Viviana Corich, Alessio Giacomini

**Affiliations:** ^1^ Department of Agronomy, Food, Natural Resources Animal and Environment (DAFNAE) University of Padova Padova Italy; ^2^ Interdepartmental Centre for Research in Viticulture and Enology (CIRVE) University of Padova Conegliano Italy; ^3^ Bioactive Compounds and Carbohydrates (BIOCARB) Research Group, Department of Food Science and Technology Universidade Federal de Viçosa Viçosa Minas Gerais Brazil; ^4^ Department of Land, Environment, Agriculture and Forestry (TESAF) University of Padova Legnaro Italy

**Keywords:** acetic acid bacteria, antioxidant activity, phenolic compounds, SCOBY, texture profile

## Abstract

This study investigated kombucha as an innovative functional starter for sourdough breadmaking. A kombucha‐based sourdough was prepared and reached technological maturity in just 5 days, significantly faster than conventional spontaneous fermentation. Its performance was compared against a traditional artisanal sourdough control. Bread quality was comprehensively evaluated through physicochemical, structural, and sensory assessments. Results demonstrate that kombucha breads exhibit specific volume, crumb structure, texture, and staling behavior comparable to traditional sourdough bread. Sensory analysis confirmed high overall acceptability and similarity between the products. Notably, kombucha breads displayed significantly higher total phenolic content and antioxidant capacity with respect to the traditional artisanal sourdough bread. These findings highlight kombucha as a highly promising alternative starter capable of accelerating sourdough production while enhancing the potential functional and nutritional properties of bread.

## Graphical abstract

Kombucha can be used as a microbial starter to accelerate sourdough fermentation, reducing maturation time from 10–14 days to about 5 days. The resulting bread maintains comparable texture, structure, and sensory qualities to traditional sourdough while offering enhanced functional benefits, including higher phenolic content and antioxidant capacity.

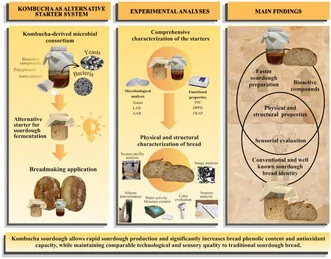

## 1 Introduction

In recent years, growing consumer awareness of the strong link between nutrition and health has driven the demand for foods that offer not only nutritional value but also additional physiological and functional benefits. Consequently, the diversity and quality of food products have significantly evolved to meet these new expectations. This growing interest has led to the development and recognition of functional foods, as products capable of exerting beneficial effects on one or more target functions in the body, beyond adequate nutritional effects (Diplock et al. 2000). Among fermented products receiving considerable attention within this category, kombucha represents a particularly interesting example. Kombucha is a mildly acidic and slightly sweet, fermented beverage traditionally obtained from sugared black or green tea, although alternative substrates such as fruit‐ and plant‐based infusions have also been explored (Cardoso et al. 2020; Anantachoke et al. 2023). Its fermentation is driven by a Symbiotic Culture Of Bacteria and Yeasts (SCOBY) under aerobic conditions, leading to the production of a wide range of bioactive compounds, including organic acids, polyphenols, vitamins, amino acids and minerals. Owing to its complex biochemical composition and the presence of beneficial microorganisms, kombucha has been associated with several biological activities, such as antioxidant, antimicrobial and anti‐inflammatory effects, supporting its classification as a functional food (Onsun et al. 2025). Due to these properties, many studies have recently investigated the role of kombucha microbiota in the production of metabolites of nutritional interest (Ghion et al. 2025). However, few studies are focused on the use of kombucha as a microbial starter for the production of other fermented products, such as cheese, yogurt or bread (Vukić et al. 2021; Makvandi et al. 2021; Kilmanoglu et al. 2024).

Among these staple foods, bread has represented a primary component of human diets for centuries and is traditionally produced through the fermentation of cereal‐based doughs. While baker's yeast ( *Saccharomyces cerevisiae* ) remains the primary leavening agent in industrial breadmaking (De Vuyst et al. 2017), increasing attention has been devoted to fermentation strategies aimed at improving both the nutritional and functional properties of bread. In this context, the incorporation of alternative or complementary microorganisms has gained considerable interest. Beyond conventional baker's yeast, lactic acid bacteria and non‐*Saccharomyces* yeasts are widely employed to modulate fermentation kinetics, organic acid production, flavor development, and bioactive compound availability (Pérez‐Alvarado et al. 2022; Sadeghi et al. 2025). Among these approaches, sourdough fermentation represents one of the most established and complex biotechnological systems in bakery science (Corsetti and Settanni 2007). Sourdough is widely described as a mixture of flour and water, spontaneously fermented by lactic acid bacteria and yeasts, with acidification and leavening capacities (Gobbetti 1998). However, the spontaneous development of a mature traditional sourdough is a notoriously time‐consuming process that requires continuous back‐slopping. The search for novel microbial consortia capable of accelerating this process while enhancing the technological performance and functional attributes of bread has therefore become a relevant research focus within cereal science (Coda et al. 2014).

In this context, the use of kombucha‐derived microbial consortia as an alternative starter for sourdough fermentation represents a novel and largely unexplored approach. Rather than being directly incorporated into dough, kombucha can be employed as a microbial inoculum for the development of a sourdough starter, which can subsequently be used for breadmaking. This strategy allows the complex symbiotic culture of bacteria and yeasts naturally present in kombucha to adapt to a cereal‐based matrix and establish a stable sourdough ecosystem. Such an approach may significantly accelerate sourdough maturation while simultaneously influencing the technological, sensory, and potential functional properties of the final bread product. Recent studies have explored kombucha in breadmaking either as an ingredient in bread formulations (Kilmanoglu et al. 2024) or as a microbial starter for the development of sourdough together with baker's yeast as the main leavening agent (Chen et al. 2025). However, these approaches were mainly based on short‐term fermentations or on the use of strains isolated from kombucha. In contrast, the present study investigated the direct use of liquid kombucha as a naturally complex inoculum for the development of a refreshed sourdough system through consecutive backslopping steps and its use in the breadmaking process.

Physicochemical, structural and sensory analyses revealed that kombucha bread retains most of the characteristics of traditional sourdough bread; instead, phenolic content and antioxidant capacity resulted significantly higher in kombucha bread.

## 2 Materials and Methods

### 2.1 Tea and Kombucha Preparation

Tea and kombucha were prepared according to the procedure of Cardoso et al. (2020) with slight modifications. To obtain 1 L of kombucha, tea was prepared by transferring 12 g of black tea leaves ( *Camellia sinensis* L., Revas Ltd., Brazil) to 1 L of sterile water at 70°C and incubated for 60 s. Subsequently, the infusion was filtered using a clean cotton cloth and allowed to cool down to room temperature.

Then, 10% (v/v) kombucha and 3% (w/v) SCOBY from a previous preparation maintained in the lab (and periodically refreshed for more than 2 years) were used as inoculum, once the tea cooled down to room temperature. SCOBY was purchased from the Departamento de Tecnologia Alimentos, Universidade Federal de Viçosa, Viçosa, Brazil. Jars (Bormioli Luigi, Parma, Italy) were covered with a clean cotton cloth and let to ferment for 7 days at 23°C. Everyday, pH was measured using a bench pH meter (HANNA Instruments, USA). At the end of the fermentation period, the newly produced SCOBY was removed and weighed, and the kombucha was then stored at 4°C.

### 2.2 Flour Microbial Analysis

The wheat flours used in the present study were Petra 6384 (Molino Quaglia, Vighizzolo d'Este Italy) for sourdough and Petra 5037 (Molino Quaglia, Vighizzolo d'Este, Italy) for bread preparation. According to the manufacturer's specification, Petra 6384 contained protein 15.5%, ash 0,5%, wet gluten 49.0%, fat 2.0%, carbohydrate 68.0%, and dietary fiber 2.7%; Petra 5037 contained protein 13.0%, ash 0.6%, wet gluten 45.0%, fat 2.0%, carbohydrate 68.5%, and dietary fiber 2.8%.

The microbial load of the flour used for sourdough preparation was determined as described by Celano et al. (2016) by dissolving 1 g of flour in 9 mL of buffered peptone water (10 g/L peptone from meat, 5 g/L sodium chloride, 1.5 g/L monopotassium phosphate, 3.5 g/L disodium hydrogen phosphate) and plating serial dilutions on plate count agar medium (Merck, Darmstadt, Germany) using the pour plate method. Plates were then incubated at 30°C for 48 h.

### 2.3 Sourdough Preparation

The process for kombucha‐based sourdough (KS) preparation takes 5 days and it is summarized in Figure S1. One hundred grams of flour were mixed in a beaker with 100 mL of kombucha until a homogenous dough consistency was achieved and then incubated at 23°C for 24 h. On the second day, the refreshment was performed by mixing 100 g of fermented dough with 100 g of flour and 50 mL of tap water, followed by incubation at 23°C for 24 h. On the third and fourth days, the same procedure was repeated, reducing the amount of water to 45 mL. After 24 h from the last refreshment, the kombucha sourdough was considered mature. The gradual reduction of dough yield during KS preparation was adopted to progressively adapt the fermentation system from the initial liquid kombucha inoculum to a more stable and manageable dough consistency suitable for Type I sourdough maintenance and breadmaking applications. This stepwise adjustment also allowed a gradual transition toward the hydration conditions typically used in artisanal sourdough propagation (Minervini et al. 2014). The pH was monitored daily using a bench‐top pH meter by immersing the electrode directly into the dough. Three independent KS propagations were prepared.

As a traditional sourdough (TS) we used an existing and daily refreshed artisanal Type I sourdough. KS preparation and TS propagation and daily refreshments were performed according to common artisanal backslopping practices described for Type I sourdough fermentation systems (De Vuyst and Neysens 2005).

### 2.4 Breadmaking Process

KS and TS were used as the leavening agents for bread production. Bread doughs were prepared according to common artisanal sourdough breadmaking practices (Cauvain 2015), using 600 g of wheat flour, 500 g of commercial mineral water, 200 g of sourdough (corresponding to approximately 33% of flour weight), and 12 g of sodium chloride. Doughs were kneaded in a mechanical kneading machine for about 20 min until a homogeneous structure was achieved. Bulk fermentation was carried out at 28°C until the dough volume approximately doubled, monitored using graduated fermentation containers. Then the dough was divided and shaped to form four individual loaves (ca 325 g each) that were placed into proofing baskets and subjected to fermentation at 4°C for 12 h. Baking was performed on a preheated refractory stone at 250°C for 40 min. Then loaves were cooled at room temperature on a wire rack to allow moisture equilibration before further analyses.

Three independent kombucha sourdough propagations were used to prepare three separate kombucha bread dough batches. Each dough batch was subsequently divided into the four individual loaves used for the analytical determinations. Conversely, traditional sourdough bread was produced starting from a single established artisanal sourdough maintained in our laboratory using one dough batch subsequently divided into four loaves.

### 2.5 Microbiological Analysis of Kombucha

Microbiological analyses of kombucha were performed at the beginning and at the end of the fermentation period for the following microbial groups, according to commonly adopted culture‐dependent methods for kombucha microbiota characterization (Marsh et al. 2014; Villarreal‐Soto et al. 2018). Acetic acid bacteria were counted on GYC (Glucose Yeast Calcium Carbonate) agar medium (glucose 50 g/L, yeast extract 10 g/L, calcium carbonate 5 g/L, agar 20 g/L); presumptive lactic acid bacteria were enumerated on MRS (de Man Rogosa and Sharpe) agar medium. Both media were supplemented with 100 mg/L cycloheximide. Yeasts were enumerated on YPD (Yeast Extract Peptone Dextrose) agar medium (glucose 20 g/L, yeast extract 10 g/L, peptone 20 g/L, agar 20 g/L), supplemented with 100 mg/L of chloramphenicol. Antimicrobial stock solutions were filter sterilized using 0.22 μm sterile filters (Sartorius, Göettingen, Germany). GYC and YPD plates were incubated at 30°C for 96 and 72 h, respectively, while MRS plates were incubated at 37°C for 96 h under anaerobic conditions using jars equipped with AnaeroGen 2.5 L oxygen‐absorbing sachets (Thermo Fisher Scientific, Waltham, MS, USA). All commercial culture media, reagents, and antimicrobial agents were purchased from Merck (Darmstadt, Germany).

### 2.6 Microbiological Analyses of Sourdough

Ten grams of sourdough were mixed with 90 mL of 0.9% sodium chloride solution in a Seward Stomacher 400 (Seward Ltd., UK) for 2.5 min at 260 rpm. Yeasts and acetic acid bacteria were enumerated on YPD and GYC agar media, respectively, under the same conditions used for kombucha, while lactic acid bacteria were enumerated on MRS5 medium (Lattanzi et al. 2013) which, according to the authors, allows better discrimination of lactic acid bacteria in sourdough. Plates were incubated at 30°C for 96 h under anaerobic conditions.

### 2.7 Sample Preparation for Chemical Analyses

KS and TS samples were collected when the sourdough was ready for breadmaking, whereas bread samples were taken 2 h after baking, once room temperature was reached. The extraction protocol of Czaja et al. (2020) was used with slight modifications. Samples were frozen at −20°C, subsequently lyophilized using the Modulyo 4 K freeze‐dryer (Edwards, UK), ground to fine powder using a steel mortar and then stored in airtight containers at −20°C. For the analyses, 1 g of lyophilized sample was mixed with 5 mL of 80% (v/v) water:methanol solution and then treated with an Elmasonic P sonicator (Elma Schmidbauer GmbH, Singen, Germany) for 15 min. Afterwards, tubes were vortexed for 10 min and centrifuged for 10 min at 5000 rpm (NEYA 16 R, Bormac, Carpi, Italia). The extraction process was repeated twice, and the supernatants were combined. This procedure produced 10 mL of extract from 1 g of lyophilized sample.

Liquid kombucha was directly used for phenolic content and antioxidant assays following appropriate dilution when necessary.

### 2.8 Antioxidant Activity and Phenol Analysis

#### 2.8.1 2,2‐Diphenyl‐1‐Picrylhydrazyl (DPPH) Radical Scavenging Assay

DPPH assay was performed following the Ebrahimi et al. (2022) method with slight modification. A 0.1 mM DPPH stock solution was prepared by dissolving DPPH powder (Merck, Darmstadt, Germany) in 96% ethanol. To generate a standard calibration curve, a 1 mM Trolox stock solution was prepared by dissolving Trolox powder (Merck, Darmstadt, Germany) in 70% ethanol solution and then diluted with the same solution to concentrations from 2 to 80 μg/mL.

To perform the test, 200 μL of sample were added to 1800 μL of DPPH solution. A negative control was prepared by mixing DPPH solution with ethanol 70%. The mixtures were incubated in the dark at room temperature for 30 min and absorbance was then measured at 517 nm using a spectrophotometer (Onda UV‐21, Giorgio Bormac S.r.l. Carpi, Italy).

Kombucha and tea samples were diluted to 1:25 with ethanol 96%, while KS, TS, and bread samples were not diluted. The radical scavenging capacity was calculated using a regression equation and expressed as mg of Trolox equivalent (TE)/100 mL for kombucha, and mg TE/g lyophilized samples for sourdough and bread.

#### 2.8.2 Ferric Reducing Antioxidant Power (FRAP) Assay

The FRAP assay was performed according to Benzie and Strain (1996) with slight modifications. To prepare the FRAP working solution, 300 mM acetate buffer (3.1 g/L sodium acetate trihydrate, 16 mL/L glacial acetic acid, pH 3.6), 10 mM 2,4,6‐Tri‐(2‐pyridyl)‐s‐triazine in 40 mM hydrochloric acid and 20 mM iron (III) chloride solutions were mixed at a 10:1:1 (v/v/v) ratio and brought to 37°C. Trolox was used for the calibration curve using a 0.1 mg/mL stock solution that was diluted in water to concentrations ranging from 0 to 60 μg/mL. Aliquots of 200 μL of sample were added to 1800 μL of FRAP solution and incubated for 30 min at 37°C. Absorbance was measured at 593 nm.

Kombucha and tea samples were diluted in distilled water at a 1:80 ratio, while TS and KS, and bread extracted samples were not diluted. The results were expressed in mg TE/100 mL for kombucha and tea and in mg TE/g lyophilized samples for sourdough and bread.

#### 2.8.3 Total Phenolic Content (Folin–Ciocalteu Assay)

The total phenolic content (TPC) of kombucha was measured using the Folin–Ciocalteu method, according to Singleton et al. (1999) with slight modifications. A standard calibration curve was created using gallic acid (GA). Distilled water was used to dissolve the GA at different concentrations, from 50 to 500 mg/L. Working solutions were prepared by mixing 100 μL of sample, 6000 μL of distilled water, 500 μL of Folin–Ciocalteu reagent (Supelco, Merck, Darmstadt, Germany), and 1500 μL of 20% sodium carbonate solution. Finally, 1900 μL of distilled water were added to bring the volume to 10 mL. Mixtures were incubated for 2 h in the dark at room temperature, and absorbance was measured at 760 nm.

Kombucha and tea samples were diluted 1:2 with distilled water while TS, KS, and bread samples were not diluted. Results were expressed in mg GAE/100 mL for tea and kombucha and mg GAE/g lyophilized samples for sourdough and bread.

### 2.9 Physical and Structural Characterization of Bread

#### 2.9.1 Volume Determination

Loaf volume was determined using the rapeseed displacement Approved Method (AACC 10–05.01). Four loaves of each type of bread (TB and KB) were gently placed in a graduated volumetric container previously filled to a reference mark with rapeseeds. Then the loaf was carefully removed and the volume of the remaining rapeseed recorded. Loaf volume was calculated as the difference between the initial and final rapeseed volumes and the specific volume (cm^3^/g) was obtained by dividing the loaf volume by its corresponding weight.

#### 2.9.2 Water Activity and Moisture Content

Water activity and moisture analyses were performed at two different time points: immediately after cooling following baking (T0) and after 48 h (T48). During storage, bread slices were kept unwrapped and exposed to air in an incubator at 21°C and 60% relative humidity.

The water activity (a_w_) of bread crumb and crust was determined using a LabMaster‐a_w_ instrument (Novasina AG, Lachen, Switzerland) at 25°C. Before each analytical session, the instrument was calibrated with the manufacturer standard salt solutions. All samples were collected from freshly sliced loaves as disks of about 2.5 cm diameter and 1 cm thickness. Crumb samples were obtained from the center of the loaf, whereas crust samples were taken from the upper external surface. Due to the relatively thick crust developed under the adopted artisanal sourdough baking conditions, the potential inclusion of crumb material during crust sampling was considered negligible. All T0 measurements were performed immediately after sampling, while T48 samples were stored under aforementioned conditions.

Moisture content of T0 and T48 crumb and crust samples was determined gravimetrically according to the Official Methods of Analysis of AOAC International No. 925.10 (AOAC 925.10) and results expressed as g of H_2_O per g of dry matter of sample (g H_2_O g^−1^ DM).

#### 2.9.3 Evaluation of Color

Color measurements were performed on bread loaves using a colorimeter (Chroma Meter CR‐300, Konica‐Minolta, Milan, Italy) operating in the CIE Lab* color space. Six technical replicates measurements were collected from each individual loaf at multiple independent sampling points on both crumb and crust. Before measurements, the colorimeter was calibrated using a standard white reference plate with known Lab*. Measurements were performed under standardized conditions using the CIE (Commission Internationale de l'Eclairage) D65 standard illuminant and a 10° standard observer angle. Color parameters were expressed as L* (lightness), a* (red–green axis), and b* (yellow–blue axis). Chroma (C*), Hue angle (H*), and Browning Index (BI) were calculated as previously described by Giannone et al. (2018) and Ficco et al. (2023).

#### 2.9.4 Image Analysis

Digital image analysis was employed to assess crumb structure according to Guan et al. (2023), with minor adjustments. The crumb structure was comprehensively evaluated through digital image analysis. One cm‐thick slices were meticulously collected from the center of the loaves and positioned on a flatbed scanner (Epson Expression 10000XL, Regent Instruments, Canada), with a certificated ruler placed adjacent to the crumb to serve as a dimensional reference. Scanning was performed using the software VueScan version 9.7.98 (Hamrick Software, Phoenix, Arizona, USA) with the following settings: professional mode, scanning mode Flatbed, media size to A4 (210 × 297 mm), color scan, 24 bit RGB, 1200 dpi resolution, and saved as TIFF format and subsequently processed using ImageJ2 version 1.4 (National Institutes of Health, Bethesda, MD, USA).

For each sample, a standardized subregion of 60 × 60 mm was selected from the center of the slice to ensure consistency and representativeness of the analysis. The porosity evaluation was rigorously performed through threshold‐based image binarization via Otsu algorithm (Otsu 1979) to quantify the proportion of void space in the crumb structure. The following parameters were analyzed: total number of cells, total area of cells (mm^2^), average area of cells (mm^2^), perimeter, circularity, solidity, maximum pore diameter (mm), minimum pore diameter (mm), Feret angle, and Aspect Ratio (AR). Circularity was used as an indicator of pore regularity and shape uniformity, whereas solidity was calculated as the ratio between pore area and convex hull area, providing an estimate of pore compactness and boundary regularity (Onipe et al. 2020).

#### 2.9.5 Texture Profile Analysis

Texture profile analysis (TPA) was performed using a TA.Xtplus Texture Analyzer (Stable Micro Systems, London, UK) equipped with a 50 kg load cell and controlled by Texture Exponent software (Stable Micro Systems, London, UK), following a previously described double‐compression approach for food texture characterization (Lomolino et al. 2025). For both traditional (TB) and kombucha (KB) breads, four central slices (with uniform 2‐cm thickness) were collected from four loaves. Texture analysis was performed for both T0 and T48 samples. Prior to analysis, bread slices were positioned on the analyzer platform and compressed using an HDP/PFS probe (Stable Micro Systems, London, UK). The test consisted of a double compression cycle performed at a crosshead speed of 2 mm/s, with a pre‐test force of 20 g. Samples were compressed to 30% of their original height, followed by probe withdrawal and a second compression cycle. Force‐time curves were recorded at a data acquisition rate of 250 points per second. The following TPA parameters were calculated: hardness (maximum force during the first compression), cohesiveness (ratio of the area under the second compression curve to that of the first, A2/A1), springiness (ratio of recovered deformation to induced deformation), gumminess (product of hardness and cohesiveness), chewiness (product of gumminess and springiness), and resilience (ratio of the area during the withdrawal phase of the first compression to the area of compression, ΔA1/A1), according to the definitions reported by Peleg (2019).

### 2.10 Sensory Analysis

The sensory evaluation was conducted by a panel of 30 untrained judges, equally distributed between males and females, with ages ranging from 25 to 55 years. All panelists evaluated both KB and TB during the same session, since the loaves were prepared at the same time. The attributes that were evaluated referred to crumb and crust visual appearance, crumb and crust taste, crumb odor, and texture. Responses were collected in binary form (presence/absence) for each attribute. However, it should be noted that the binary sensory approach adopted in this study represents a preliminary screening method and may not detect subtle intensity differences among sensory attributes. Future studies should include more robust sensory methodologies, such as hedonic scaling or quantitative descriptive analysis (QDA), to obtain a more detailed characterization of consumer perception and sensory profiles.

### 2.11 Statistical Analysis

All statistical analyses were performed using R software (version 4.2.3; R Foundation for Statistical Computing, Vienna, Austria). Results are reported as mean ± standard deviation of biological replicates, and statistical significance was set at *p* < 0.05.

For kombucha bread, independent biological replicates corresponded to separate sourdough propagations and breadmaking batches, whereas multiple loaves obtained from the same dough preparation were used as technical replicates for analytical determinations. Traditional sourdough bread was produced from a single dough batch subsequently divided into multiple loaves.

Microbiological counts obtained for flour, kombucha, and sourdough samples were log‐transformed prior to analysis and expressed as log CFU g^−1^ or mL^−1^. Differences among microbial populations were evaluated using one‐way analysis of variance (ANOVA), followed by Tukey's post hoc test when significant effects were detected.

Antioxidant properties were analyzed according to the experimental design of each dataset. For kombucha samples, comparisons between tea and kombucha were performed using non‐parametric tests due to the limited number of biological replicates (*n* = 3 per group). Antioxidant properties of sourdough and bread were analyzed using two‐way ANOVA with fermentation type (traditional vs. kombucha) and processing stage (sourdough vs. bread) as fixed factors, including their interaction. When significant effects were detected, pairwise comparisons were performed using Tukey's post hoc test.

Specific volume and crumb image analysis parameters were compared between traditional kombucha breads using Welch's *t*‐test.

Water activity, moisture content, color parameters, and texture profile analysis were analyzed using linear mixed‐effects models to account for repeated measurements on the same loaf. Fermentation type, storage time (T0 vs. T48), and/or bread portion (crumb vs. crust) were included as fixed effects depending on the analyzed variable, while loaf was included as a random intercept. When significant effects were detected, pairwise comparisons were performed using Tukey‐adjusted post hoc tests.

Binary sensory data were analyzed using McNemar's test to evaluate paired differences between traditional and kombucha breads for each sensory attribute.

## 3 Results and Discussion

### 3.1 Flour Microbial Analysis

Analysis of total viable counts of three different batches of flour purchased in different periods gave a mean value of 3.52 ± 0.16 log CFU/g, without any significant statistical difference among batches, indicating a quantitative constant microbial composition of the raw material. This result aligns with data obtained by Cardoso et al. (2019), that reported an aerobic plate count ranging from 3.43 to 5.42 log CFU/g in different flours, including refined wheat flours.

### 3.2 Preparation of Kombucha

#### 3.2.1 Kombucha Characteristics

The kombucha and SCOBY selected for this study were chosen since they underwent extensive characterization in recent years. Specifically, the microbial consortium composition and stability were examined over a 1‐year period of monthly refreshments (Ghion 2025). Furthermore, nutritional and functional properties have been documented through in vitro assays (Lacerda et al. 2024), simulated digestion trials (Ghion et al. 2025), and several in vivo studies involving both animal models (Costa et al. 2022) and obese human volunteers (Bonifácio et al. 2025; de Campos Costa et al. 2025; Fraiz, Bonifácio, et al. 2024a, 2024b; Fraiz, Costa, et al. 2024).

#### 3.2.2 pH Evolution

The starting sweetened tea had a slightly acidic pH of 5.92. The addition of SCOBY and kombucha inocula brought the pH down to 3.97 ± 0.01 which highlights the drastic effect of the inoculum. Over the 7‐day fermentation period, the pH of kombucha significantly decreased to 3.20 ± 0.06 (Figure 1A). The gradual decrease reflects the increasing accumulation of organic acids, which contributes to the kombucha antimicrobial properties. This pH decline also favors the activity of acetic acid bacteria (De Miranda et al. 2022). Sadok et al. (2025) reported that the fermentation temperature significantly affects the final pH, with 22°C indicated as the optimal condition to get a pH of 2.94 after 14 days. These findings are in line with our results, where a final pH of 3.20 was observed after 7 days of fermentation at 23°C ± 2°C. In contrast, fermentation at 17°C and 25°C resulted in too low pH values (2.55 and 2.51), respectively, that could negatively affect the subsequent sourdough production.

**FIGURE 1:**
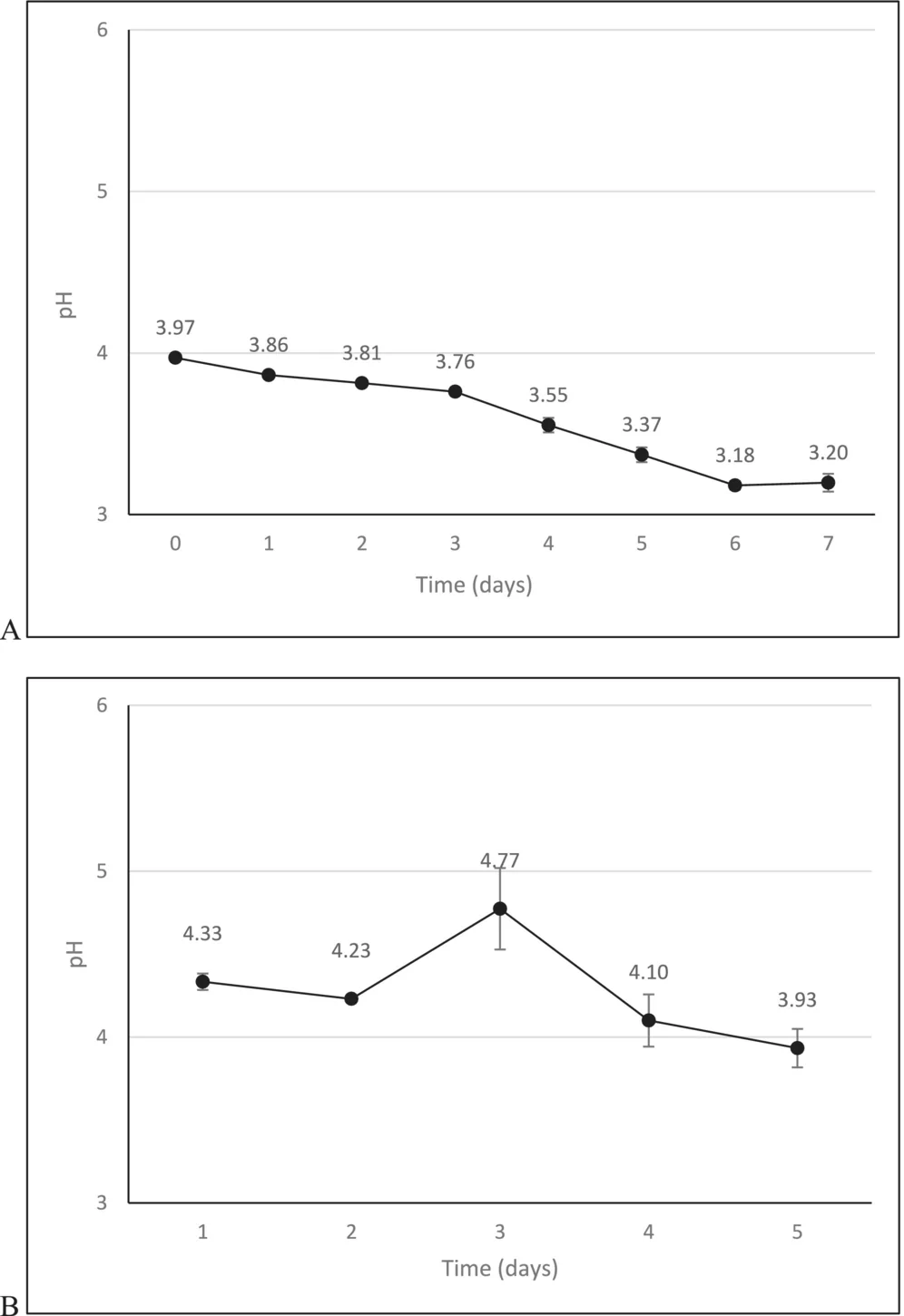
Evolution of pH during fermentation processes. (A) pH variation of kombucha during 7 days of fermentation. (B) pH variation of KS during 5 days needed for its preparation. Values represent mean ± standard deviation of three biological replicates.

#### 3.2.3 Kombucha Microbiology and Biomass Development

The kombucha 7‐days fermentation induced a significant increase of acetic acid bacteria, from 3.70 ± 0.43 to 4.44 ± 0.12 log CFU/mL. Although yeasts were largely dominant over bacteria at the beginning, they did not show a statistically significant variation (from 6.06 ± 0.08 log CFU/mL at T0 to 6.67 ± 0.23 log CFU/mL at T7). Presumptive lactic acid bacteria count was always below the detection limit (1 log cell/mL).

Several factors can affect the composition of kombucha, leading to variations in microbial counts. Comparing our results with those reported by Wang et al. (2023), several similarities and differences were observed. In both studies, acetic acid bacteria exhibited a significant increase during kombucha fermentation. However, while yeast increase was significant in their study, it was not statistically significant in ours. Their initial yeasts and acetic acid bacteria counts were similar (3.08 log CFU/mL) and increased markedly by the end of a 14‐day fermentation period, with acetic acid bacteria being dominant, contrary to what we found in our study. This reflects the influence of the difference in the protocol used: while their fermentation lasted for 14 days at 22°C with a greater inoculum (20% v/v), our study employed a shorter 7‐day fermentation at 23°C ± 2°C with a 10% (v/v) inoculum. In general, optimal fermentation temperatures range from 18°C to 26°C. An optimal fermentation length is also crucial to obtain an acceptable flavor and taste and prevents the potential risks of high levels of acid production (Jayabalan et al. 2014).

The increase of acetic acid bacteria also determined an increase of the SCOBY biomass, which grew from an initial weight of 15 g, used as inoculum, to 22.87 ± 2.7 g at T7 (i.e., 35% increase), due to the development of the cellulose matrix produced by bacterial activity.

#### 3.2.4 Antioxidant Properties of Kombucha

The antioxidant properties of kombucha were evaluated by comparing DPPH radical scavenging activity, FRAP, and TPC with those of the corresponding tea infusions. Analyses were performed on three independent biological batches for both tea and kombucha. As shown in Table 1, no significant differences were observed between tea and kombucha for DPPH, FRAP, and TPC values. This indicates that kombucha fermentation did not affect the overall phenolic content nor the antioxidant properties of the starting tea. This result is consistent with previous observations (Ghion et al. 2025) reporting that the antioxidant profile of kombucha is strongly influenced by the characteristics of the initial tea infusion, rather than by the fermentation process. From a technological perspective, the preservation of antioxidant properties during kombucha fermentation is relevant, as it suggests that the fermentation process does not determine degradation of phenolic compounds or antioxidant power. In the context of the present study, this result supports the use of kombucha as a fermentation starter primarily for its microbial and fermentation‐related properties, including dough acidification and leavening capacity, while antioxidant activity appears mainly linked to the tea.

**TABLE 1:** DPPH, FRAP, and TPC activity of tea and kombucha.

	Tea	Kombucha
DPPH	123.22 ± 27.91^a^	115.40 ± 19.30^a^
FRAP	486.95 ± 53.12^a^	466.86 ± 40.68^a^
TPC	85.45 ± 1.90^a^	89.88 ± 5.57^a^

*Note:* Results are expressed as mg TE/100 mL of kombucha for DPPH and FRAP and as mg GAE/100 mL of kombucha for TPC. Results represent mean values of biological replicates (*n* = 3). Different letters denote statistically significant differences between groups (*p* < 0.05).

### 3.3 Sourdough Microbial Analysis and pH

Microbiological analysis of sourdough samples (Table 2) compared TS with KS. On day 5 of KS production, the yeast population was not significantly different from that of TS. Indeed, it was reported by Kilmanoglu et al. (2024) that the number of yeasts may vary depending on the species present in the dough. They observed a slight decrease in the yeast counts during their kombucha sourdough fermentation, concluding that the species in the kombucha microbiota could not adapt to the sourdough environment. On the other hand, our KS showed a significant 2‐log increase of lactic acid bacteria, indicating a more favorable environment for their growth. Remarkable, acetic acid bacteria, that were below the detection limit in TS, developed abundantly in KS, following their introduction with the kombucha used as inoculum, indicating flour to be a good substrate for their growth. Finally, at maturity, kombucha dough contained an equilibrated presence of the three main microbial components, all of them reaching the same order of magnitude.

**TABLE 2:** Yeast counts on YPD, lactic acid bacteria on MRS5 and acetic acid bacteria on GYC of traditional and kombucha sourdough.

	Traditional sourdough	Kombucha sourdough	
YPD	7.34 ± 0.05^a^	8.06 ± 0.23^a^	
MRS5	6.61 ± 0.18^a^	8.19 ± 0.07^b^	
GYC	< 1	8.18 ± 0.04	

*Note:* Data are expressed as mean ± standard deviation. Different letters indicate significant statistical differences (*p* < 0.05). Values are expressed as log CFU/g of sourdough. All measurements were performed in triplicate.

The pH of KS was monitored throughout the fermentation period to evaluate the progress of the acidification process (Figure 1B). From the initial value of 4.33 ± 0.05, following the addition of kombucha, the mature dough showed a pH drop of ca. 0.4 units, reflecting acid production from a successful fermentation process. In fact, the optimal pH of a sourdough should range from 3.4 to 4.9 (Arora et al. 2021). A temporary increase in pH was observed on day 3. This fluctuation may be associated with refreshment‐related dilution effects, flour buffering capacity, and temporary shifts in microbial metabolic activity during the adaptation of the kombucha‐derived consortium to the sourdough environment (De Vuyst and Neysens 2005; Minervini et al. 2014).

Type I sourdoughs generation requires repeated backslopping cycles for more than a week before reaching microbiological and technological stability, when they are obtained just from whey flour and water (Savic 2009; Minervini et al. 2014). In contrast, the use of liquid kombucha as inoculum introduces an already active fermentative microbial consortium, likely contributing to the rapid establishment of physical (e.g., volume increase) (Figure S2) and microbiological characteristics suitable for breadmaking within only 5 days.

### 3.4 Evolution of Phenolic Content and Antioxidant Capacity During the Breadmaking Process

As reported in Table 3, KS exhibited a significantly higher antioxidant capacity and phenolic content than traditional sourdough across all assays, indicating that the fermentation type strongly in KS compared to TS, suggesting an enhanced release or transformation of redox‐active compounds during fermentation. Sourdough fermentation has been widely reported to promote the liberation of phenolic compounds bound to the cereal matrix through microbial enzymatic activity, particularly associated with lactic acid bacteria, resulting in increased antioxidant potential (Katina et al. 2005; Hur et al. 2014). The higher values observed in KS may therefore be attributed to the metabolic activity of a more complex microbial consortium, whose synergistic interactions between yeasts and acetic acid bacteria could further intensify these bioconversion processes, as previously reported for kombucha fermentations (Villarreal‐Soto et al. 2018). Baking represented a critical processing step leading to a general reduction in phenolic content and antioxidant capacity in all breads, reflecting the impact of thermal processing on bioactive compounds. Thermal processing is known to affect the stability and extractability of phenolic compounds in cereal‐based products. Several studies have reported that baking may induce degradation and structural modifications of phenolic acids, particularly in bound forms, leading to changes in antioxidant activity and overall phenolic content in the final baked product (Skrajda‐Brdak et al. 2019; Alfeo et al. 2020). Despite these processing‐related losses, kombucha bread retained significantly higher FRAP and TPC values than traditional bread, indicating that the higher initial levels established during fermentation were better preserved in the final product. No significant differences in DPPH activity were observed between breads, highlighting known differences among antioxidant assays and the complexity of antioxidant behavior in fermented and baked matrices. While TPC and FRAP assays showed consistent trends across fermentation and baking stages, DPPH activity displayed less pronounced differentiation in the final product, a discrepancy commonly reported in the literature and attributable to the different chemical mechanisms underlying each assay, rather than inconsistencies in the experimental results (Prior et al. 2005).

**TABLE 3:** DPPH, FRAP and TPC results of traditional and kombucha sourdough and of the respective breads.

	DPPH		FRAP		TPC	
	Traditional	Kombucha	Traditional	Kombucha	Traditional	Kombucha
Sourdough	12.73 ± 0.96^a^	16.44 ± 0.55^b^	154.975 ± 1.88^b^	231.34 ± 3.25^d^	1.61 ± 0.03^c^	2.30 ± 0.01^d^
Bread	13.66 ± 0.72^a^	13.10 ± 0.46^a^	134.82 ± 1.55^a^	187.31 ± 13.12^c^	0.47 ± 0.01^a^	0.66 ± 0.01^b^

*Note:* Data are expressed as mean ± standard deviation. Kombucha bread analyses were performed on three independent breadmaking batches, whereas traditional bread samples were obtained from multiple loaves produced from a single dough batch. Different letters indicate statistically significant differences (*p* < 0.05) among samples within each analytical method (DPPH, FRAP, and TPC), considering both fermentation type (traditional and kombucha) and processing stage (sourdough and bread). Results are reported as mg TE/g of lyophilized sample for DPPH and FRAP, and as mg GAE/g of lyophilized sample for TPC.

The higher phenolic content and antioxidant power observed in kombucha sourdough and bread likely reflected the combined contribution of tea‐derived phenolic compounds introduced through the liquid kombucha inoculum and their subsequent preservation during sourdough fermentation and baking. Since no significant differences were observed between tea and kombucha before sourdough preparation, these results suggest that the antioxidant‐related properties were mainly associated with the tea matrix rather than being generated during kombucha fermentation.

### 3.5 Physical and Structural Characteristics of Bread

#### 3.5.1 Bread Specific Volume

Bread specific volume was measured at T0. TB and KB showed values of 2.456 ± 0.141 cm^3^/g and 2.621 ± 0.239 cm^3^/g, respectively. These results are expressed as mean ± standard deviation of the four replicates for each type of bread (TB and KB). No statistically significant differences were detected, indicating that the use of kombucha as sourdough starter does not affect loaf expansion under the adopted breadmaking conditions. These results are in contrast with previous reports that described kombucha breads with reduced loaf volume and more compact crumb structure (Kilmanoglu et al. 2024). From a technological point of view, this result is relevant, as bread volume is a key quality parameter, reflecting level of gas production, dough rheology, and ability of the gluten network to retain fermentation gases.

Sourdough bread volume is influenced by multiple factors, including microbial population composition, fermentation kinetics, dough acidification level, and flour characteristics. However, previous studies have shown that different sourdough starters, characterized by distinct microbial consortia, can yield breads with comparable loaf volume when dough formulation and fermentation conditions are properly adjusted. De Vuyst et al. (2017) highlighted that physical outcomes such as loaf volume may converge despite microbiological differences.

Similarly, Coda et al. (2014) reported that the technological performance of sourdough, including gas retention and bread volume, depends more on overall fermentative balance and dough structure rather than on the specific starter culture, especially when complex microbial communities are involved. In this context, the absence of significant differences observed in the present study suggests that our kombucha‐derived microbial consortium was able to establish fermentative kinetics comparable to that of traditional sourdough.

The use of non‐conventional starters has been previously explored in sourdough breadmaking, with several studies reporting technological equivalence rather than superiority when alternative microbial sources are employed. For example, Corsetti and Settanni (2007) emphasized that starter diversity does not necessarily translate into improved loaf volume, but rather into changes in aroma, acidity, and microbial stability, while maintaining comparable physical properties of bread.

#### 3.5.2 Moisture and Water Activity of Crumb and Crust

In the present study, both water activity and moisture were strongly influenced by the bread portion (crumb or crust) and storage time of the bread, whereas no significant differences were observed between breads produced with traditional and kombucha sourdough.

Crumb showed consistently higher values of water activity compared to crust, as expected, and a decrease during storage from T0 to T48. Moreover, no significant differences were observed between breads produced with traditional and kombucha sourdough (Table 4).

**TABLE 4:** Moisture content and water activity of crumb and crust of KB and TB at T0 and T48.

	Moisture				Water activity			
	T0		T48		T0		T48	
	Kombucha	Traditional	Kombucha	Traditional	Kombucha	Traditional	Kombucha	Traditional
Crumb	0.95 ± 0.012^c^	0.94 ± 0.02^c^	0.329 ± 0.039^b^	0.355 ± 0.112^b^	0.910 ± 0.003^cd^	0.912 ± 0.001^d^	0.801 ± 0.004^bc^	0.843 ± 0‐025^b^
Crust	0.2 ± 0.037^a^	0.21 ± 0.068^a^	0.15 ± 0.01^a^	0.18 ± 0.03^a^	0.806 ± 0.006^b^	0.820 ± 0.016^b^	0.628 ± 0.040^a^	0.640 ± 0.044^a^

*Note:* Data are expressed as mean ± standard deviation. Kombucha bread analyses were performed on three independent breadmaking batches, whereas traditional bread samples were obtained from multiple loaves produced from a single dough batch. Different letters indicate statistically significant differences (*p* < 0.05) among samples within moisture and a_w_, considering both fermentation type (traditional and kombucha), bread portion (crumb and crust), and storage time (T0 and T48). Results are reported as g H_2_O/g of bread (dm) for moisture.

Moisture content differed significantly between crumb and crust and decreased during storage, particularly in the crumb fraction. Also in this case, the type of sourdough starter did not significantly influence moisture values at any time point.

These findings contrast with the heterogeneous results reported in the literature for kombucha‐based breads. Kilmanoglu et al. (2024) reported higher moisture in kombucha breads, whereas other studies have shown the opposite trend, with reduced moisture compared to conventional bread. For instance, bread formulated with kombucha tea as a liquid phase exhibited significantly lower crumb moisture than control samples, likely due to differences in fermentation dynamics and water binding within the dough matrix (Kaya and Asir 2022). Such discrepancies among studies may be attributed to differences in kombucha formulation, fermentation time, dough hydration, and baking conditions, all of which strongly influence water retention and redistribution during baking. The lack of significant differences observed here supports the view that moisture retention in kombucha‐based breads is not an intrinsic effect of kombucha fermentation itself, but rather a formulation‐ and process‐dependent variable.

#### 3.5.3 Bread Color Evaluation

Instrumental color analysis was performed by measuring several parameters, including lightness (L*), chromatic coordinates (a* and b*), chroma (C*), hue angle (H*), and browning index (BI) (Table S1). Results revealed that color was primarily linked to the bread region considered (crumb vs. crust), while the type of sourdough starter (traditional vs. kombucha) did not result in statistically significant differences for any of the evaluated color parameters. The pronounced differences between crumb and crust agree with well‐established thermal and physicochemical phenomena occurring during baking, since crust is subjected to intense dehydration and higher temperatures, which promote non‐enzymatic browning transformations, primarily Maillard reactions and caramelization, leading to reduced lightness and increased browning‐related indices. On the other hand, the crumb retains higher moisture levels and is subjected to milder thermal exposure, resulting in lighter color attributes and lower browning intensity. The absence of statistically significant differences between TB and KB evidences that the use of kombucha as an alternative fermentation system does not substantially modify color development during baking. This finding is technologically relevant, as color represents a primary quality attribute directly perceived by consumers and strongly associated with product identity and acceptability (Purlis 2010). Comparable results have been reported from previous studies investigating kombucha‐based sourdough breads. Kilmanoglu et al. (2024) observed that the use of kombucha‐derived starters did not induce marked changes in crumb and crust color. Similarly, Kaya and Asir (2022) reported non‐statistically different effects of kombucha enrichment on color parameters of bread.

#### 3.5.4 Image Analysis of Crumb Structure

Image analysis of crumb structure (Table 5) did not evidence significant differences between traditional and kombucha breads in terms of pore count, total pore area, mean pore size, and pore area fraction. These parameters describe the overall porosity of the crumb, indicating that the number of gas cells, their cumulative area, and their contribution to the total crumb surface were comparable between the two bread types. Representative cross‐sectional images of the crumb, together with the corresponding regions of interest and binarized images used for quantitative analysis, are shown in Figure 2. Visual inspection supports the quantitative findings, as both breads exhibited a well‐developed and homogeneous crumb structure without marked differences in pore density or distribution. The similarity observed at macroscopic and microscopic levels further reinforces the absence of statistically significant differences in global porosity‐related parameters. Significant differences were instead detected for selected pore shape descriptors. Circularity values were higher in kombucha breads compared to traditional sourdough breads, indicating a more regular and rounded pore geometry. Higher circularity values are generally associated with more uniform gas cell expansion. No significant differences were instead observed for solidity values, indicating comparable pore compactness and boundary regularity between the two bread types. Feret diameter analysis revealed significant differences between the two bread types. This parameter provides information on pore elongation, and the lower Feret diameters observed in kombucha breads suggest slightly smaller and less elongated pores, which is consistent with the higher circularity values. Additionally, a significative difference was detected in Feret angle. Although this parameter is considered less important than size‐ and shape‐ related descriptors, the observed difference suggests a modest alteration in pore orientation patterns, potentially linked to differences in dough rheology or gas cell stabilization mechanisms between the two fermentation systems.

**TABLE 5:** Crumb image analysis of traditional bread (TB) and kombucha bread (KB).

Parameter	TB	KB	Significance
Count (*n*)	663 ± 65	684 ± 163	ns
Total area (mm^2^)	666.17 ± 91.29	661.26 ± 34.46	ns
Average pore size (mm^2^)	1.01 ± 0.17	1.00 ± 0.23	ns
Pore area (% ROI)	41.60 ± 5.70	41.31 ± 2.16	ns
Perimeter (mm)	3.42 ± 0.26	3.21 ± 0.06	ns
Circularity (0–1)	0.479 ± 0.014	0.514 ± 0.006	*
Solidity (0–1)	0.768 ± 0.007	0.776 ± 0.004	ns
Feret max (mm)	0.91 ± 0.04	0.82 ± 0.03	*
Feret min (mm)	0.49 ± 0.02	0.44 ± 0.03	*
Feret angle (°)	97.41 ± 2.22	79.82 ± 6.28	**

*Note:* Data are expressed as mean ± standard deviation. Kombucha bread analyses were performed on three independent breadmaking batches, whereas traditional bread samples were obtained from multiple loaves produced from a single dough batch. Significance levels are indicated as follows: ns, *p* ≥ 0.05.

* *p* < 0.05.

** *p* < 0.01.

**FIGURE 2:**
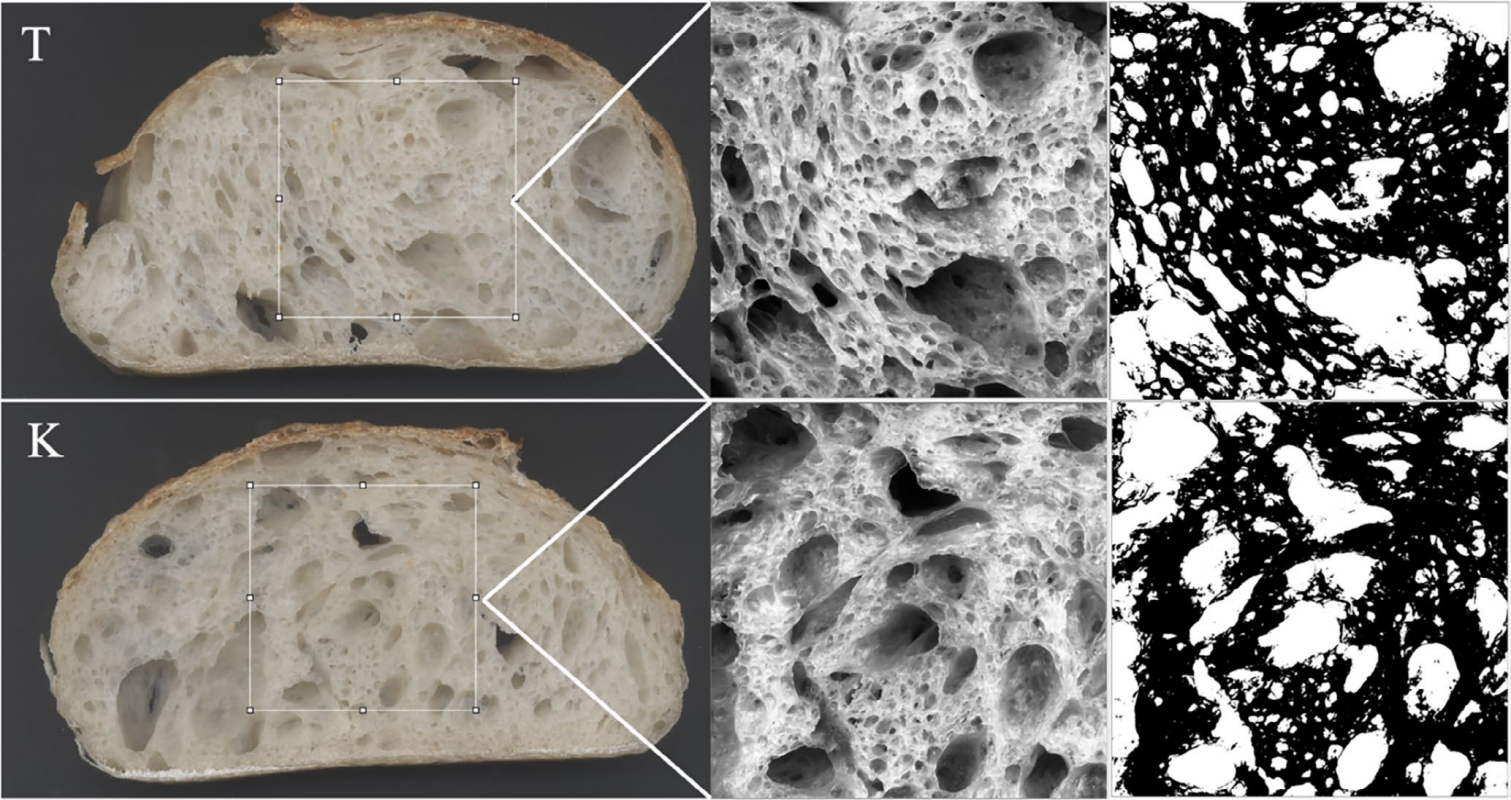
Workflow used for crumb image analysis of breads produced with traditional (T) and kombucha (K) sourdough. From left to right: Cross‐sectional image of the bread slice with the selected Region of Interest (ROI), magnified grayscale image of the ROI, and corresponding binarized image used for quantitative analysis of pore morphology.

Overall, image analysis indicates that kombucha‐based sourdough fermentation preserves the overall crumb architecture while inducing subtle modifications in pore geometry. This interpretation is consistent with the absence of significant differences in bread specific volume and with the comparable textural properties observed between traditional and kombucha breads. Similar observations have been reported in previous studies investigating alternative or mixed microbial starters in sourdough breadmaking. Kilmanoglu et al. (2024) stated that the use of kombucha‐derived microbial consortia does not lead to any alteration in crumb structure when compared with traditional sourdough products.

#### 3.5.5 Texture Profile Analysis

Texture profile analysis of KB and TB at T0 and T48 is reported in Table 6. Results reveal that storage time had a significant effect on several mechanical parameters, whereas the type of sourdough starter did not induce significant differences in texture evolution. In both breads, hardness increased markedly after 48 h. The significant increase in hardness observed for both fermentation systems reflects the typical crumb firming associated with bread staling. A similar trend was observed for gumminess and chewiness. These changes indicate a progressive increase in energy required to deform and disintegrate the crumb during mastication, consistent with moisture redistribution and starch retrogradation processes occurring during storage. Cohesiveness decreased significantly over time in both breads, and this reduction suggests a weakening of internal structural bonds within the crumb matrix, a phenomenon commonly reported during bread staling. In contrast, resilience was statistically unchanged across fermentation types and storage times, indicating that the elastic recovery of the crumb was largely preserved despite the increase in firmness. Springiness results of the two fermentation types were not statistically different, suggesting that kombucha fermentation did not substantially alter the elastic behavior of the crumb during storage. The comparable behavior observed in traditional and kombucha breads indicates that kombucha‐based sourdough supports a staling process similar to that of conventional sourdough breads. This finding is consistent with previous studies investigating kombucha‐derived starters in breadmaking, which reported that the resulting breads exhibited textural properties comparable to those obtained using traditional sourdough fermentation (Kilmanoglu et al. 2024).

**TABLE 6:** Texture profile analysis of traditional bread (TB) and kombucha bread (KB) at T0 and T48.

	T0		T48	
	TB	KB	TB	KB
Hardness (g)	1497.32 ± 98.31^a^	1309.52 ± 81.52^a^	30457.39 ± 3536.59^b^	28763.49 ± 5319.97^b^
Resilience	0.37 ± 0.02^a^	0.36 ± 0.01^a^	0.43 ± 0.20^a^	0.29 ± 0.02^a^
Cohesiveness	0.76 ± 0.02^b^	0.75 ± 0.02^b^	0.54 ± 0.10^a^	0.57 ± 0.04^a^
Gumminess	1131.96 ± 51.37^a^	979.49 ± 66.65^a^	16428.86 ± 2784^b^	16481.60 ± 3037.91^b^
Springiness	0.89 ± 0.03^b^	0.87 ± 0.01^ab^	0.77 ± 0.09^a^	0.78 ± 0.06^ab^
Chewiness	1013.39 ± 75,62^a^	853.43 ± 60.41^a^	12733.14 ± 3193,89^b^	12849.11 ± 2038.38^b^

*Note:* Data are expressed as mean ± standard deviation. Kombucha bread analyses were performed on three independent breadmaking batches, whereas traditional bread samples were obtained from multiple loaves produced from a single dough batch. Different letters indicate significant differences (*p* < 0.05).

### 3.6 Sensory Analysis of Bread

Sensory analysis showed a high level of similarity between traditional and kombucha breads (Figure S3). Out of the 72 evaluated sensory attributes, no significant differences were observed for 69 descriptors (*p* ≥ 0.05), indicating similar perception between products across most visual, olfactory, gustatory, and textural attributes. Significant differences were detected only for three attributes. Specifically, TB was perceived different from KB for a higher odor of fresh bread and slightly sour taste perception, while KB differed for softer crumb. Although no significant differences were detected instrumentally for hardness values, sensory evaluation evidenced a slightly softer crumb perception for kombucha bread. This apparent discrepancy may be related to the fact that oral texture perception is influenced not only by mechanical resistance but also by saliva interaction, moisture release, lubrication, and crumb breakdown during mastication, which are not fully captured by instrumental TPA measurements (Szczesniak 2002).

The overall sensory similarity between traditional and kombucha breads observed in this study aligns with recent findings on kombucha‐driven sourdough fermentation. Kilmanoglu et al. (2024) stated that the sensory acceptability of kombucha breads did not differ markedly from that of traditional bread. Likewise, Supasil et al. (2022) demonstrated that bread obtained from sourdough made with fermented extracts of Asian pears and Assam tea leaves does not affect the overall sensory acceptability.

Finally, some limitations of the present study should be acknowledged. First, while kombucha bread was produced from three independent biological replicates, traditional sourdough bread was obtained from a single dough batch divided into multiple loaves, which limits the assessment of batch‐to‐batch variability for the traditional control. However, the use of a highly stable and historically well‐characterized artisanal starter mitigated this limitation, ensuring a robust and reproducible benchmark. Second, the sensory evaluation was restricted to a preliminary screening based on a binary approach with untrained panelists, which may not capture subtle intensity variations or detailed sensory profiles. Nevertheless, this approach successfully established that kombucha bread possesses high overall acceptability and closely shares the primary sensory traits of traditional sourdough bread, confirming that no off‐flavors or major defects were introduced. Future investigations using Quantitative Descriptive Analysis (QDA) will be beneficial to fully characterize consumer perception. Lastly, the functional evaluation of antioxidant properties and phenolic content relied exclusively on spectrophotometric in vitro assays. While these assays conclusively demonstrated a significantly higher retention of bioactive compounds in kombucha sourdough and bread, further research incorporating simulated gastrointestinal digestion and bioavailability models is required to definitively validate the in vivo nutritional and health advantages of this novel functional product.

## 4 Conclusions

This study successfully demonstrates the potential of kombucha as an innovative and functional starter for sourdough breadmaking. Utilizing kombucha significantly accelerates the fermentation process, yielding a mature sourdough in just 5 days, which is remarkably faster than the time required for traditional spontaneous fermentation. It should be emphasized that this accelerated establishment observed in the present study was compared to the generation of a new sourdough system starting from just flour and water.

The introduction of the kombucha microbial consortium into the dough established a stable fermentative ecosystem comprising yeasts, acetic acid bacteria, and lactic acid bacteria. However, the present study relied exclusively on culture‐dependent enumeration methods. Future investigations based on molecular approaches would be necessary to better characterize microbial succession and interactions during sourdough fermentation.

Compared with recent studies on kombucha‐enriched breads, the present work specifically investigated liquid kombucha as an inoculum for sourdough development through consecutive refreshments, rather than only as a bread ingredient or a short‐term fermentation component. This distinction is relevant because the objective was not only to evaluate bread enrichment but also to assess whether kombucha could support the preparation of a sourdough suitable for breadmaking while maintaining bread quality.

Kombucha sourdough and the resulting bread exhibited significantly higher total phenolic content and increased in vitro antioxidant capacity compared to traditional sourdough products. This indicates that the presence of kombucha‐derived tea components resulted in higher levels of phenolic compounds and in vitro antioxidant capacity in the final sourdough and bread products.

Crucially, this enrichment did not compromise the technological or sensory quality of the final product. The kombucha‐driven fermentation preserved the overall crumb architecture. Furthermore, kombucha bread demonstrated physicochemical and structural properties—including specific volume, moisture, color, and crumb porosity—that were completely comparable to conventional sourdough bread. Sensory evaluation further validated these findings, revealing a high level of similarity and acceptability, with no significant differences detected in the vast majority of the evaluated attributes.

Overall, the combined acceleration of sourdough development and the significant enhancement of antioxidant properties position kombucha as a highly promising alternative starter culture for the production of bioactive‐enriched bakery products. To fully validate the potential bioactive enrichment of KOMBREAD, further in vivo studies are required to assess the bioavailability and physiological impact of its enriched bioactive profile upon human consumption.

## Author Contributions

**Manuel Chinello:** methodology, investigation, formal analysis, writing – original draft. **Ragy Nader Ragheb Tawfik:** investigation, formal analysis, writing – original draft. **Elisa Canazza:** methodology, investigation, formal analysis. **Chiara Nadai:** data curation, visualization, writing – review and editing. **Frederico Augusto Ribeiro de Barros:** conceptualization, resources. **Giovanna Lomolino:** conceptualization, data curation. **Anna Lante:** conceptualization, resources. **Viviana Corich:** conceptualization, writing – review and editing. **Alessio Giacomini:** project administration, funding acquisition, supervision, writing – review and editing.

## Funding

This study was funded in part by the University of Padua (Progetto di Ateneo, BIRD228113) “Fermentation of plant products and by‐products to obtain functional foods and bioactive compounds”, by the Italian Ministry of Foreign Affairs and International Cooperation (Progetti di grande rilevanza, cod. BR22GR06) and by the Veneto Region POR FESR 2021–2027 Programme (CUP D19J24000690007).

## Ethics Statement

The sensory evaluation conducted in this study was formally approved by the competent ethics committee of the University of Padova, DAFNAE Department (Protocol No. 0003267, 26 June 2025; UOR: D160000; Classification: II/4). All participants voluntarily agreed to participate in the study and provided informed consent prior to the sensory evaluation. Participation was voluntary and sensory data were collected anonymously. The study involved the sensory evaluation of food products and did not involve invasive procedures or the collection of sensitive personal data.

## Conflicts of Interest

The authors declare no conflicts of interest.

## Supporting information


**Table S1:** Color parameters of crumb and crust from TB and KB.
**Figure S1:** Flow chart of step‐by‐step protocol of kombucha‐based sourdough preparation.
**Figure S2:** Volume increase of kombucha sourdough. (1) Immediately after the third refreshment; (2) 24 h after the third refreshment.
**Figure S3:** Absolute frequency of positive responses for all sensory descriptors evaluated in TB and KB. Each bar represents the number of panelists reporting the presence of a given attribute. Descriptors are grouped according to sensory category.

## Data Availability

The data that support the findings of this study are available from the corresponding author upon reasonable request.
