# Supplementary material for: KOMBREAD, Kombucha as a Functional Starter for Sourdough Bread: Physicochemical Properties, Microbiological Characterization, and Quality Evaluation
Source: Food Sci Nutr. 2026 Sep 30;14(10):e72433. doi: 10.1002/fsn3.72433 (PMC13624710; doi:10.1002/fsn3.72433)
Supplement: Supplementary file 1 [file FSN3-14-e72433-s001.docx]

**SUPPLEMENTARY MATERIAL**

**Table S1.** Color parameters of crumb and crust from TB and KB.

|  | **Crumb** | | **Crust** | |
| --- | --- | --- | --- | --- |
|  | **Traditional** | **Kombucha** | **Traditional** | **Kombucha** |
| **L*** | 68.03 ± 0.70^b^ | 68.17 ± 1.23^b^ | 55.65 ± 2.00^a^ | 58.38 ± 1.72^a^ |
| **a*** | 1.76 ± 0.08^b^ | 1.58 ± 0.10^b^ | 11.35 ± 0.83^a^ | 11.04 ± 1.18^a^ |
| **b*** | 18.37 ± 0.26^b^ | 17.91 ± 0.38^b^ | 27.72 ± 1.86^a^ | 28.46 ± 2.56^a^ |
| **C*** | 18.45 ± 0.26^b^ | 17.98 ± 0.38^b^ | 30.03 ± 1.89^a^ | 30.58 ± 2.80^a^ |
| **H*** | 5.47 ± 0.21^b^ | 5.03 ± 0.24^b^ | 22.47 ± 1.73^a^ | 21.26 ± 0.56^a^ |
| **BI** | 32.73 ± 0.94^b^ | 31.56 ± 1.31^b^ | 82.61 ± 8.37^a^ | 79.88 ± 12.28^a^ |
| Data are expressed as mean ± standard deviation. Kombucha bread analyses were performed on three independent breadmaking batches, whereas traditional bread samples were obtained from multiple loaves produced from a single dough batch. Different superscript letters within the same row indicate statistically significant differences (*p* < 0.05). | | | | |

Day 1 100 g flour + 100 mL kombucha Incubate 23°C, 24 h

Day 2 100 g dough + 100 g flour + 50 mL water Incubate 23°C, 24 h

Day 3 100 g dough + 100 g flour + 45 mL water Incubate 23°C, 24 h

Day 4 100 g dough + 100 g flour + 45 mL water Incubate 23°C, 24 h

Day 5 Sample collection, downstream analysis or breadmaking

**Fig. S1.** Flow chart of step-by-step protocol of kombucha-based sourdough preparation.

**Fig. S2.** Volume increase of kombucha sourdough. 1) Immediately after the third refreshment; 2) 24 h after the third refreshment.

Crumb texture

|  | TB | KB |
| --- | --- | --- |
| Soft | 21 | 23 |
| Moist | 22 | 19 |
| Dry | 0 | 0 |
| Compact | 5 | 4 |
| Aerated | 13 | 13 |
| Spongy | 24 | 20 |
| Crumbly | 0 | 0 |
| Elastic | 21 | 19 |

Crumb odour

|  | TB | KB |
| --- | --- | --- |
| Slightly sour | 12 | 17 |
| Fresh bread | 15 | 8 |
| Yeasty | 11 | 12 |
| Buttery | 1 | 2 |
| Musty | 1 | 0 |
| Toasted | 1 | 0 |
| Fermented | 10 | 17 |
| Lactic | 8 | 12 |
| Neutral | 8 | 4 |
| Intense | 6 | 5 |

Crumb taste

|  | TB | KB |
| --- | --- | --- |
| Salty | 0 | 3 |
| Intense | 4 | 4 |
| Sweet | 3 | 4 |
| Umami | 3 | 2 |
| Neutral | 13 | 14 |
| Fermented | 13 | 10 |
| Bitter | 1 | 0 |
| Slightly sour | 14 | 15 |

Crumb mouthfeel

|  | TB | KB |
| --- | --- | --- |
| Compact | 6 | 5 |
| Moist | 22 | 20 |
| Soft | 13 | 20 |
| Pasty | 5 | 5 |
| Dry | 1 | 1 |
| Aerated | 6 | 12 |
| Crumbly | 0 | 1 |
| Melting | 10 | 7 |
| Gummy | 14 | 15 |

Crust appearance

|  | TB | KB |
| --- | --- | --- |
| Matte | 7 | 6 |
| Thick crust | 1 | 3 |
| Glossy | 2 | 2 |
| Regular | 5 | 9 |
| Uniform colour | 2 | 5 |
| Variegated | 20 | 20 |
| Irregular | 10 | 11 |
| Dark brown | 3 | 2 |
| Burnt | 1 | 1 |
| Light brown | 18 | 12 |
| Pale | 3 | 1 |
| Thin Crust | 21 | 24 |

Crust taste and aroma

|  | TB | KB |
| --- | --- | --- |
| Bitter | 0 | 1 |
| Acidic | 3 | 5 |
| Yeasty | 5 | 8 |
| Nutty | 2 | 3 |
| Flat | 8 | 6 |
| Toasted | 9 | 15 |
| Malty | 5 | 3 |
| Salty | 3 | 2 |
| Sweet | 1 | 2 |
| Slight sour | 18 | 13 |
| Umami | 1 | 1 |
| Short flavour | 8 | 8 |
| Wheaty | 6 | 10 |
| Persistent | 8 | 6 |
| Caramelised | 0 | 2 |

**Fig. S3.** Absolute frequency of positive responses for all sensory descriptors evaluated in TB and KB. Each bar represents the number of panelists reporting the presence of a given attribute. Descriptors are grouped according to sensory category.
